# Tracing the Past: Renewing Life Narratives Through Robots

**DOI:** 10.1093/geroni/igaa057.3407

**Published:** 2020-12-16

**Authors:** Anna Ueda, Hideyuki Takahashi

**Affiliations:** 1 St. Cloud State University, Minneapolis, Minnesota, United States; 2 Osaka University, Toyonaka, Osaka, Japan

## Abstract

The study explores the effectiveness and efficacy of using robotics in clinical settings to facilitate Life Review. Life Review is a process in which subjects retrospectively analyze major life events with a conversation partner in order to find meaning and to synthesize a narrative. In this experiment, Life Review was conducted with 5 elderly subjects and two types of partners: a human and a robot. The partners utilized a set of trigger questions to review past events with their subjects. Two sequences of Life Review, each comprising four sessions, were completed. Four sessions involved a human partner, and four involved a robot partner. The recorded correspondences in Life Review were transcribed, and the utterances of the participants with the two partners were compared and analyzed qualitatively. This preliminary study was the first attempt to explore the benefits of conducting Life Review with robotic conversation partners. The results showcased distinct differences between a human partner and a robotic partner. Specifically, subjects in sessions with a human partner showed stronger awareness of generational gaps between the human partner rather than the robotic partner. In contrast, sessions with a robotic partner included more universally transmissive values. The outcome suggests Life Review with robots can potentially provide elderly patients greater safety and comfort in telling their unique life narratives. The usage of robotic partners in Life Review provides a promising and novel research area into improving and re-imagining mental health access and outcomes for patients.

